# Cross-Analytical Strategies to Tackle “Medicines in Disguise” Presented as Food Supplements, a New Threat for Human Health

**DOI:** 10.3390/molecules30061372

**Published:** 2025-03-19

**Authors:** Judith Nzoughet Kouassi, Chouaha Bouzidi, Béatrice Nicolai, Farah Ben Jamaa, Annabelle Dugay, Jérôme Langrand, Dominique Vodovar, Pascal Houzé, Laurence Labat, Bruno Mégarbane, Cinzia Bocca, Pascal Reynier, Nicolas Guiblin, Sylvie Michel, Xavier Cachet

**Affiliations:** 1 UFR de Pharmacie, Faculté de Santé, Université Paris Cité, CNRS, Cibles Thérapeutiques et Conception de Medicaments, UMR 8038, F-75006 Paris, France; judith.nzoughet-kouassi@u-paris.fr (J.N.K.); chouaha.bouzidi@u-paris.fr (C.B.); beatrice.nicolai@u-paris.fr (B.N.); farahbenjamaa17@gmail.com (F.B.J.); annabelle.dugay@u-paris.fr (A.D.); sylvie.michel@parisdescartes.fr (S.M.); 2 Laboratoire SPMS, Centrale Supelec, Université Paris Saclay, Plateau de Moulon 3 rue Joliot-Curie, F-91192 Gif-sur-Yvette, France; nicolas.guiblin@centralesupelec.fr; 3 Centre Antipoison et de Toxicovigilance de Paris, Hôpital Fernand-Widal APHP, Inserm UMR-S 1144, F-75010 Paris, France; jerome.langrand@aphp.fr (J.L.); dominique.vodovar@chu-rouen.fr (D.V.); 4 Laboratoire de Toxicologie, Hôpital Lariboisière APHP, Inserm UMR-S 1144, F-75010 Paris, France; pascal.houze@aphp.fr (P.H.); laurence.labat@aphp.fr (L.L.); 5 Réanimation Médicale et Toxicologique, Hôpital Lariboisière APHP, Inserm UMR-S 1144, F-75010 Paris, France; bruno.megarbane@lrb.aphp.fr; 6 Faculté de Santé, Institut MITOVASC, Université d’Angers, UMR CNRS 6015, INSERM U1083, F-49000 Angers, France; cinzia.bocca@univ-angers.fr (C.B.); pascal.reynier@univ-angers.fr (P.R.)

**Keywords:** plant-based food supplement, adulteration, medicine in disguise, liquid chromatography (LC), high-resolution mass spectrometry (HRMS), nuclear magnetic resonance (NMR), X-ray powder diffraction (XRPD)

## Abstract

Plant-based food supplements (FS) of doubtful traceability have now emerged as a new threat to human health. Food supplements adulterated with pharmaceutical ingredients are considered “medicines in disguise” by regulatory authorities, which is a sub-category of falsified medicines. In the context of illegal manufacture and trade, as well as in the absence of an official phyto- and/or pharmacovigilance system, emergency departments and poison control centers constitute a early warning system for detecting ingested suspect FS. In the present investigation, we set up efficient workflows for the systematic characterization of adulterated plant-based FS in the context of an original local early warning alert system (i.e., FalsiMedTrack) involving an emergency department, a poison center, and academic analytical chemistry laboratories. Fit-for-purpose cross-analytical methods were employed, including sophisticated methods such as liquid chromatography coupled to high-resolution mass spectrometry, nuclear magnetic resonance, X-ray powder diffraction, as well as the most accessible and affordable HPLC method with UV/DAD detection. The strategy was applied successfully to typical cases of suspect plant-based health products, i.e., sample incriminated in patients experiencing side effects and herbal products currently commercialized for their “amazing health benefits”. The samples contained active pharmaceutical ingredients, including diclofenac, piroxicam, dexamethasone 21-acetate, and sibutramine. We provided evidence of “medicines in disguise” presented as food supplements, which raises concerns about their quality and safety.

## 1. Introduction

According to the European Union (EU) General Food Law Regulation (EC) No 178/2002 and consolidated version of 1 July 2022, food supplements are considered foodstuffs. They are defined as concentrated sources of nutrients or other substances with a nutritional or physiological effect that are marketed in various forms (e.g., pills, tablets, capsules, gummies, powder, and liquids). A wide range of nutrients and other ingredients might be present in food supplements, including, but not limited to, vitamins, minerals, amino acids, essential fatty acids, fiber, and various plants and herbal extracts. The purpose of food supplements is to provide beneficial nutritional supplements in addition to the daily diet [1,2,3].

A market analysis reported that the global market for vitamins and dietary supplements has grown at a compound annual growth rate (CAGR) of 6.3% between 2014 and 2018, mainly driven by food supplements [4].

This market was estimated to reach USD 139.38 billion in 2024 and is expected to be worth USD 173.69 billion by 2029. Following the COVID-19 pandemic, the market has seen an increase in the demand for food supplements, especially in plant-based FS, which are expected to boost the market growth in the next five years [5].

The growing interest in plant-based food supplements is driven by the intent of improving overall health, losing weight, building muscle, slowing down the aging process, mental well-being, improving memory, and better sexual performance, among others. The increase in the consumption of food supplements has simultaneously led to an increase in the introduction of falsified supplements into the market, which pertains to the manufacture and sale of non-authorized products, the use of low-cost non-standard materials or/and not tested or approved by regulatory authorities [4]. In this context, the Internet plays a significant role in the spreading of adulterated food supplements.

It is worth recalling that any substance with exclusively pharmacological properties, thus intended for medicines, cannot be used as an ingredient for a food supplement. A food supplement may resemble a medicine in its presentation, e.g., in the form of capsules or pills; nevertheless, a food supplement is not a medicine. It has no therapeutic action and should never be taken as a substitute for medical treatment [1].

Medicine contains an active pharmaceutical ingredient (API) and excipients, each one displaying a specific role. The active ingredient corresponds generally to a compound or a mixture of compounds that can be obtained by extraction, total chemical synthesis, hemi-synthesis, or biotechnological means, with a curative or preventive action, unlike excipients, which have no pharmacological effect on the organism. In that sense, food supplements adulterated with pharmaceutical agents are considered “medicines in disguise” by regulatory authorities, which is a sub-category of falsified medicines. Currently, “medicines in disguise” refer to products not presented as medicine but containing undeclared APIs [6].

Both official control laboratories and academic researchers have reported cases of food supplement adulterations.

Back in 2007, the US Food and Drug Administration created a Dietary Supplement Database to identify adulteration with APIs. A recent review of the database pinpointed 1068 adulterations from 2007 to 2021, with sexual enhancement and weight loss dietary supplements being the most common products adulterated with APIs. Phosphodiesterase-5 inhibitors (PDE-5i), including sildenafil, tadalafil, vardenafil, avanafil, and their unapproved analogues, were present in sexual enhancement dietary supplements, whilst sibutramine, an appetite-suppressant drug removed from the market in 2010 due to cardiovascular adverse events, was the most detected adulterated API in weight loss products [7].

In Europe, the European Directorate for the Quality of Medicines and Healthcare (EDQM) coordinates the activities of the General European Official Medicines Control Laboratories (OMCL) Network (GEON), who conducted several market surveillance programs and retrospective studies from 2012 to 2019 [6,8,9]. The existing trends in the adulteration of food supplements in the EU were further supported by a recent work of Amidži’ and co-authors, who screened the EU Rapid Alert System for Food and Feed (RASFF) database for the decade 2011–2022 [10]. In addition to the presence of anabolics, PDE-5i, and sibutramine found in the analyszd samples, these investigations shed light on emerging substances, e.g., research peptides, Selective Androgen Receptor Modulators (SARMs), nootropic drugs (i.e., vincamine and vinpocetine), and cannabinoids (some of them, such as cannabidiol (CBD), are pending authorization as a novel food ingredient).

Besides official control laboratories and institutions, the scientific community continues to play a role in the fight against food supplement adulteration and particularly “medicines in disguise”. The community has been always proactive in establishing suitable detection and monitoring analytical methods. The research studies reported below are a few typical examples.

Huang et al., 2008, developed and validated a high-performance liquid chromatographic method, coupled with UV detection and electrospray ionization mass spectrometry (HPLC-UV-ESI-MS), for the simultaneous determination of the illegal additives sibutramine and its metabolite *N*-di-desmethylsibutramine in dietary supplements consumed for weight control [11]. A decade ago, Vaysse and collaborators reported a method based on diffusion-ordered spectroscopy (DOSY) ^1^H-nuclear magnetic resonance (NMR) and 3D DOSY-COSY ^1^H-NMR to analyze twenty herbal dietary supplements marketed as natural slimming products. The method allowed the detection of both active ingredients and excipients in these complex matrices. Among the 20 formulations analyzed, 2 were strictly herbal and 4 had a composition corresponding to the declared ingredients on the packaging or the leaflet. The others were all adulterated. Eight formulations contained sibutramine alone at doses ranging from 4.4 to 30.5 mg/capsule. Five formulations contained sibutramine (from 5.0 to 19.6 mg/capsule or tablet) in combination with the laxative phenolphthalein (from 4.4 to 66.1 mg/capsule), and the last formulation was adulterated with p-synephrine that possesses potential weight management effects (19.5 mg/capsule). DOSY NMR was proposed as a useful tool for the detection of unexpected adulteration [12]. In 2011, Stypułkowska et al. employed a different analytical strategy for identifying adulteration with sibutramine and its analogues in herbal dietary supplements. Their choice was X-ray powder diffraction (XRPD) and liquid chromatography (LC) with various types of detection: ultraviolet (UV), coulometric electrode array (CEA), and time-of-flight mass spectrometry (TOF-MS). It was argued that XRPD was useful as a fast and non-destructive screening method for general sample composition, which can easily discriminate between capsules containing exclusively pure herbal materials and those with some chemicals by visually examining diffraction patterns [13].

A few years later, Vaysse’s team identified a novel sildenafil analogue in an adulterated herbal supplement by employing nuclear magnetic resonance (NMR), mass spectrometry, and infrared spectroscopy (IR). iErect^®^, a new dietary supplement marketed as “100% natural” and sold over the Internet, was proved to contain thiosildenafil, a sildenafil analogue already reported as an adulterant in herbal formulations, and a new compound named depiperazinothiosildenafil, which resulted from the hydrolytic cleavage of the S–N bond of the sulfonamide group of thiosildenafil. A capsule of iErect^®^ was proved to contain a very high amount (≈220 mg) of thiosildenafil and around 30 mg of depiperazinothiosildenafil, which exposes consumers to the risk of serious side effects [14].

In an article dealing with the screening of synthetic PDE-5i, Patel and co-authors presented a comprehensive review of conventional and advanced analytical techniques to detect and characterize these adulterants [15].

A screening strategy based on UHPLC-HRMS (ultra-high performance liquid chromatography coupled with high-resolution mass spectrometry) combined with in-house MS/MS database has also garnered interest. Guo et al. in 2020 recently developed a UHPLC-quadrupole-orbitrap high-resolution mass spectrometry method for identifying antihypertensive adulterants in dietary supplements and herbal medicinal products. The approach was applied to 65 samples claimed as “all-natural” products claiming antihypertensive activity, among which 9 samples were adulterated at levels ranging from 2.8 to 27.9 mg/g [16].

The analytical strategy depends on the laboratory’s capabilities and the context of the analyses. It is often necessary to opt for cross-analytical methods to remove all barriers to the detection of falsification, since adulteration can involve various physico-chemical and pharmacotechnical aspects.

In this joined effort to tackle falsified medicines, we set up the FalsiMedTrack project. FalsiMedTrack is a collaborative network that brings together partners from universities, hospitals, and the Paris Poison Center. FalsiMedTrack is interdisciplinary and gathers the expertise of all project partners. It focuses on developing innovative means for mapping falsified medicines (including “medicines in disguise” like food supplements) and related illicit products which adversely affect human health. This is achieved by developing integrated physico-chemical analytical strategies, including LC-UV/MS, LC-HRMS/MS, NMR, and XRPD. These cross-analytical techniques are complementary, with each method providing specific information but also having limitations in the context of suspected falsified plant-based dietary supplements.

LC-MS is particularly useful for analyzing relatively polar, high molecular weight, low-volatile, or thermally unstable compounds. Combining LC with HRMS offers exact mass determination, access to chemical formulas, and allows chemical structure hypotheses. NMR, although not as sensitive as LC-MS, is a specific and powerful approach for the analysis of molecules alone or in mixtures, whilst X-ray powder diffraction (XRPD) is rapid and non-destructive and allows one to gain additional information on the solid state of pharmaceutical compounds (crystalline state, polymorphism, solvation). XRPD is therefore not systematically suitable for identifying contaminants, but it may be useful and complementary to chromatographic and NMR methods for fingerprinting and discrimination of falsified samples. The same applies to HPLC-DAD, the most accessible and affordable HPLC technique.

Finally, we illustrated our work by presenting analyses of two representative examples of herbal food supplements adulterated with one or more pharmaceutical ingredients.

## 2. Results and Discussion

**Figure 1:**
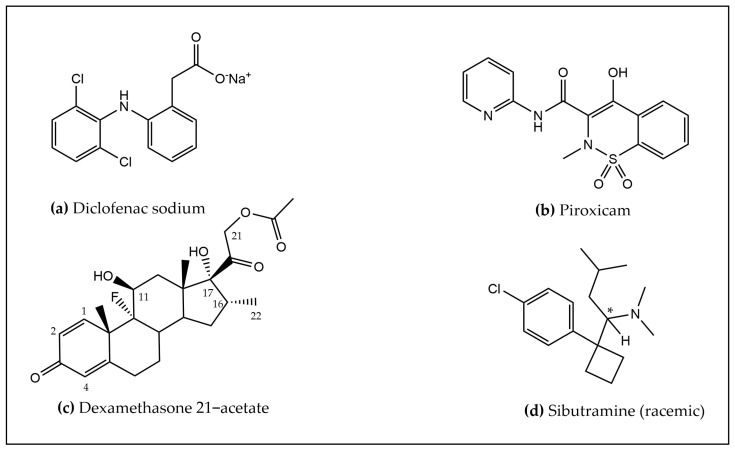
Chemical structures of the active pharmaceutical ingredients identified, i.e., (**a**) Diclofenac sodium, (**b**) Piroxicam, (**c**) Dexamethasone 21–acetate, and (**d**) Sibutramine. The asterisk symbol indicates the position of the asymmetric carbon.

In the framework of the FalsiMedTrack project, we planned out an integrated physico-chemical strategy for the characterization of adulteration in food supplements (“medicines in disguise”), based on XRPD, HPLC-UV/MS (ESI^−^), UHPLC-HRMS/MS (HESI^+^), and ^1^H NMR. The chemical structures of the active pharmaceuticals identified in these food supplements are illustrated in Figure 1.

The test materials consisted of two plant-based food supplements, namely Meng yululu capsules and Castanha da India Indiana capsules (samples B1, B2, V1, and V2).

In XRPD, capsules containing pure herbal materials are expected to exhibit a broad diffraction maximum in the 2Theta range between 9° to 30°. In contrast, samples containing a mixture of natural materials and some chemical additives will exhibit the superposition of this broad maximum and sharp Bragg peaks [13]. All the solid samples studied (Meng yululu, B1, B2, V1, and V2) exhibited additional sharp peaks characteristic of additional crystalline additives.

Considering the relative intensities of the sharp maxima and the broad background, we may conclude that samples B1, B2 contain similar crystalline compounds and the same ratio of chemical ingredients *vs* herbal material (idem for samples V1, V2). In addition, the crystalline compositions of B1/B2 seem different from samples V1/V2 and Meng yululu (see Figure S1).

### 2.1. Identification of Diclofenac in Commercial Food Supplements (Samples V1 and V2)

HPLC-UV (λ: 254 nm) chromatogram profiles of the MeOH reconstituted CH_2_Cl_2_ extracts from Castanha da India Indiana commercial food supplement samples V1, V2, B1, and B2, which confirmed XRPD observations and clearly showed that sample V1 is similar to V2, sample B1 is similar to B2, but samples V1, V2 are different from samples B1, B2. Indeed, samples V1, V2 exhibited a main peak eluting at the same retention time (26.7 min) in both food supplements’ HPLC-UV profiles, implying the presence of the same compound in both samples. Conversely, this compound seemed absent from food supplement samples B1, B2. In fact, we noticed the presence of distinct peaks eluting at different retention times, i.e., 20 min and 24 min (Figure S2).

The main peak in samples V1, V2 was subsequently identified as diclofenac (C_14_H_10_Cl_2_NO_2_) by both ESI negative ionization MS (*m*/*z* 293.9) and positive ionization mode UHPLC-HRMS/MS, as detailed below.

HPLC-UV (λ: 254 nm) ESI^−^ MS spectrum showed a [M − H]^−^ ion at *m*/*z* 293.8 for food supplement samples V1, V2, as illustrated on Figure S3.

**Figure 2:**
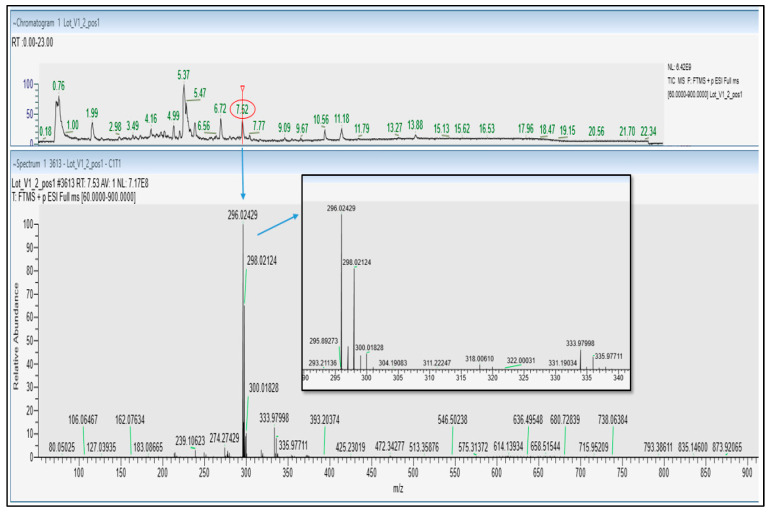
UHPLC-HESI^+^ Q Exactive Orbitrap HRMS TIC chromatogram (**top** panel) and Full MS spectrum (**bottom** panel) of plant-based food supplement Sample V1.

Positive ionization mode UHPLC-HRMS/MS analysis further confirmed the presence of diclofenac in Castanha da India Indiana samples V1, V2. The full scan MS spectra showed a [M + H]^+^ ion at *m*/*z* 296.02429 and an isotopic pattern characteristic of the presence of 2 chlorine atoms, with the monoisotopic peak A, peak A + 2, and peak A + 4 detected at *m*/*z* 296.02429 (100%), *m*/*z* 298.02124 (64%), and *m*/*z* 300.01828 (10%), respectively. This was corroborated by the similar retention time (7.52 min) and fragmentation pattern (fragment ions at *m*/*z* 278.0132, 250.0183 and 215.0494) obtained for both diclofenac analytical standard and the identified compound in samples V1, V2 (Figure 2 and Figure S4).

This is also in good agreement with MS/MS spectra recorded for diclofenac in public database such as mzCloud.

Taken together, plant-based food supplements sold online were proved to be adulterated by diclofenac, a non-steroidal anti-inflammatory drug (NSAID). Castanha da India Indiana commercial food Supplements V1, V2 were demonstrated to be “medicines in disguise” since they contain an active pharmaceutical compound, namely diclofenac.

Diclofenac is a proven, commonly prescribed nonsteroidal anti-inflammatory drug (NSAID) that has analgesic, anti-inflammatory, and antipyretic properties, and is effective in treating a variety of acute and chronic pain and inflammatory conditions. As with all NSAIDs, diclofenac exerts its action via inhibition of prostaglandin synthesis by inhibiting cyclooxygenase-1 (COX-1) and cyclooxygenase-2 (COX-2) with relative equipotency. The mechanism of action and safety profile of diclofenac was well documented by Gan [17].

### 2.2. Identification of Piroxicam and Dexamethasone 21-Acetate in the Commercial Food Supplement Samples B1, B2

As mentioned in the previous Section 2.1, HPLC-UV (λ: 254 nm) chromatograms of samples B1, B2 presented similar profiles and showed two distinct peaks of different relative intensities in both chromatograms, with eluting at 20 min and 24 min (Figure S2). We set about characterizing these unknown compounds using a cross-analytical strategy combining HPLC-UV/(ESI^−^) MS, UHPLC-HRMS/MS (HESI^+^), XRPD, and ^1^H NMR.

[M − H]^−^ ions were detected at *m*/*z* 329.9 and 433, as shown by the corresponding HPLC-UV/(ESI^−^)MS chromatograms and spectra (Figure S5).

This was in agreement with UHPLC-HRMS/MS results, where the same compounds were detected in positive ionization mode, with the [M + H]^+^ ions detected at *m*/*z* 332.06973 and 435.21777, respectively.

The fragment ions obtained for each compound initially allowed their putative identification (Figures S6–S8); the first compound was attributed to the nonsteroidal anti-inflammatory drug (NSAID) piroxicam (C_15_H_13_N_3_O_4_S) and the latter to isomeric steroidal anti-inflammatory drugs (SAID) dexamethasone and betamethasone 21-acetate (DA and BA), flunisolide, and triamcinolone acetonide (C_24_H_31_FO_6_) [18,19,20].

Finally, the structure of the SAID present in samples B1 and B2 was unambiguously determined using ^1^H NMR spectroscopy. From HPLC/UV analyses that revealed similar HPLC fingerprints, B1 and B2 samples were shown to contain the same anti-inflammatory drug but in different relative proportions; structure elucidation was further performed using Sample B2.

**Figure 3:**
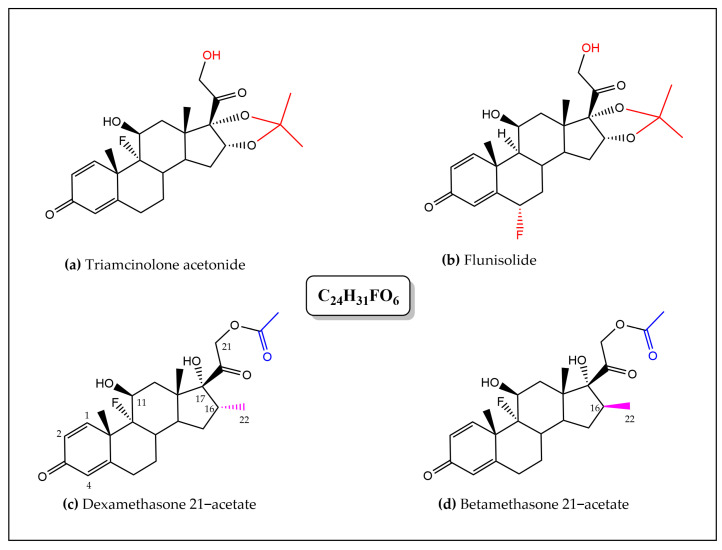
Chemical structures of the four corticosteroids suspected to be present in samples B1, B2, sharing the same chemical formula C_24_H_31_FO_6_ (colors indicate the discriminating structural features distinguished by ^1^H NMR analysis). (**a**) triamcinolone acetonide, (**b**) flunisolide, (**c**) dexamethasone 21–acetate, and (**d**) betamethasone 21–acetate.

**Figure 4:**
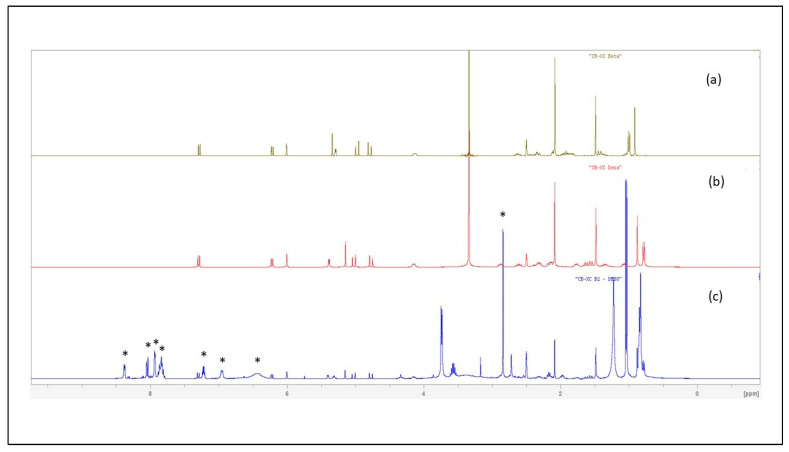
^1^H NMR spectra in DMSO-*d6* of betamethasone 21-acetate standard (**a**), dexamethasone 21-acetate standard (**b**), and of the crude CH_2_Cl_2_ extract of Sample B2 (**c**) (Asterisks indicate ^1^H NMR signals of piroxicam).

Unequivocal identification of dexamethasone 21-acetate (C_24_H_31_FO_6_) was ascertained by ^1^H NMR spectroscopy (Figure 3, Figure 4 and Figures S9–S11).

Despite inherent overlaps, different signals found in the the ^1^H NMR spectrum (in DMSO-*d6*) of the crude CH_2_Cl_2_ extract of Sample B2 could be attributed to the typical signals of a synthetic corticosteroid. Further analysis and comparison to data obtained for DA/BA reference standards allowed discrimination between the closely chemical structures of the four targeted SAID and finally an unambiguous identification of DA.

The four molecules shared several common typical signals, particularly those of the olefinic signals H-1, H-2, and H-4 of the A-ring (signals appearing at *δ* 7.29 ppm (1H, d, *J* = 10.2 Hz), 6.22 ppm (1H, dd, *J* = 10.2 Hz, *J’* = 1.8 Hz), and 6.00 ppm (1H, large s), respectively, in the spectrum of DA)), the H-11 oxygenated methine proton H-11 (appearing as a large d (*J* = 3.3 Hz) at *δ* 5.28 ppm (1H) for DA), and finally the signals of the geminal protons H21a,b of the 2-hydroxy-1-oxo-ethyl chain at C-17 (appearing two doublets 1H at *δ* 4.97 ppm (*J* = 17.7 Hz) and 4.79 ppm (*J* = 17.7 Hz) for DA). In contrast, they differed by the presence of an acetonide group in flunisolide and triamcinolone acetonide or of an acetate group in both DA and BA. The methyl signal of the acetate could be observed as a singlet 3H at *δ* 2.08 ppm in the BA and DA spectra that limited our research of the latter isomers that only differ by the stereochemistry at the C-16 (the methyl adopts a α-configuration in DA and a β-configuration in BA) [21]. Interestingly, but not surprisingly, NMR proved to be a powerful method for structure elucidation since it was able to differentiate between the two structures on the basis of the C-16 methyl chemical shift value. The signal of the methyl group was observed in the extract spectrum as a characteristic doublet (3H) resonating at *δ* 0.78 ppm (*J* = 7.2 Hz), matching the NMR signal for DA standard (The methyl signal was observed as a doublet (3H) (*J* = 7.4 Hz) at a significantly different *δ* of 1.00 ppm for BA standard). The corticosteroid was therefore unequivocally identified as DA (Figure 4 and Figures S9–S11).

**Figure 5:**
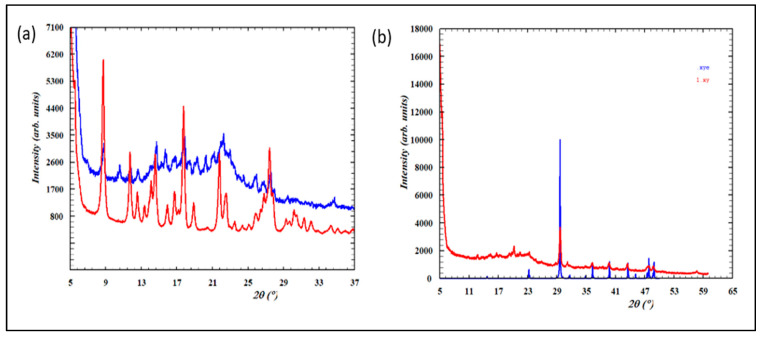
(**a**) Overlay of the experimental XRPD pattern of Sample B1 (in blue) and the pattern of the piroxicam standard (in red); (**b**) overlay of the experimental XRPD pattern of Sample V1 (in red) and the calculated pattern of a polymorph of CaCO_3_ (150-ICSD) (in blue).

XRPD data suggested that samples B1, B2 may contain piroxicam and another crystalline compound which was not identified. A rapid search in the ICSD (the Inorganic Crystal Structure Database) showed that samples V1, V2 contain a polymorph of CaCO_3_ (Figure 5), while additional Bragg peaks at low 2Theta suggested the presence of an organic crystalline compound not identified.

The ^1^H NMR spectrum of the crude CH_2_Cl_2_ extract of Sample B2 also showed the characteristic signals of piroxicam (indicated by an asterisk on Figure 4) that were found similar to reference data found in literature [22]. This observation definitively confirmed the presence of piroxicam in samples B1, B2.

Piroxicam is a nonsteroidal anti-inflammatory drug (NSAID), the first of a new class of NSAIDs known as the oxicams. It has a long half-life, which allows for once daily administration, and is a potent inhibitor of prostaglandin biosynthesis. In animal models of inflammation, piroxicam is as potent as indomethacin and, in animal models of pain, more potent than aspirin, ibuprofen, naproxen, or phenylbutazone [23]. Dexamethasone is one of the most prescribed anti-inflammatory drugs in the treatment of acute and chronic eye inflammation due to its high potency and effectiveness. It acts by binding with the corticosteroid receptors present in the human trabecular meshwork cells and inhibits phospholipase-A2 and thus prostaglandins synthesis [24]. Dexamethasone was recently highlighted as a potential therapeutic for COVID-19, due to its key indication for Acute Respiratory Disease Syndrome (ARDS) [25]. Dexamethasone 21-acetate, a derivative of dexamethasone, is an exogenous fluorinated glucocorticosteroid drug also administered for a large variety of inflammatory conditions, including inflammation in the cornea [24].

Castanha da India Indiana samples V1, V2 contained diclofenac, while Castanha da India Indiana samples B1, B2 contained piroxicam and dexamethasone 21-acetate. For food supplements deemed to be of natural origin, it is a real scam. This product is still available online and the risk associated with its consumption can be quite worrying. While seeking information on the product, we noticed two reported marketing bans concerning a manufacturer (Wanerva do Brasil Ltd.a, Sapé, Brasil) in 2015 and 2022 [26,27], and permissions reported for others [28].

### 2.3. UHPLC-HRMS/MS Identification of Sibutramine in the Slimming Herbal Food Supplements, Meng Yululu

**Figure 6:**
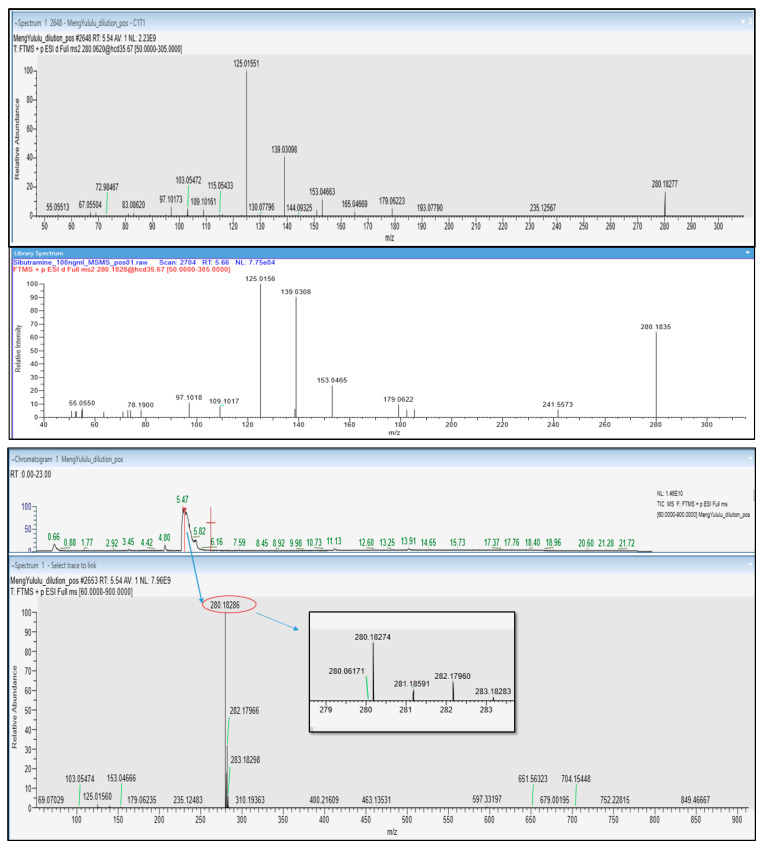
UHPLC-HESI^+^ Q Exactive Orbitrap HRMS TIC chromatogram of Meng yululu slimming food supplement, full MS spectrum, ddMS2 fragmentation spectrum of Sibutramine detected at 5.47 min, and fragmentation spectrum of Sibutramine analytical standard (**top** to **bottom** panel).

Meng yululu capsules were provided by a patient and the sample was analyzed by UHPLC-HRMS/MS. The chromatogram exhibited a very intense peak at 5.47 min, identified as sibutramine (C_17_H_26_ClN) as ascertained by the accurate *m*/*z* value (280.18274), retention time (5.47 min), Full MS, and MS/MS spectra, vs. data acquired from sibutramine analytical standard (Figure 6). The isotopic pattern of the compound in the full MS spectra is also characteristic of the presence of 1 chlorine atom, as attested by the intensity of peaks containing ^35^Cl (*m*/*z* 280.18274) and ^37^Cl (*m*/*z* 282.17960) isotopes.

Sibutramine was previously used in the treatment of obesity and type 2 diabetes. Sibutramine is a norepinephrine and serotonin reuptake inhibitor that was approved for weight management in patients who are unable to lose weight through diet and exercise alone. Sibutramine induces satiety (resulting in reduced food intake) and an increase in energy expenditure. In some patients, sibutramine increases blood pressure, pulse rate, or both, owing to its sympathomimetic effects [29]. Because of its undesirable side effects, which can lead to an increased risk of heart attacks and strokes, it was withdrawn from the market in 2010 and banned from sale in many countries, including in the EU.

The results gathered during the present work are depicted in Table 1.

In light of the current results, it is appropriate to note that health professionals and legislators have a more important role to play with, on the one hand, over-the-counter dietary supplements patients are using and, on the other hand, in improving patient awareness and safety [30]. Unlike medicines, food supplements do not have a mandatory notice to inform consumers about the safety of the product. Only brief information, such as the ingredients’ identity, is required on the label. Simply mentioning the presence of plants in food supplements can sometimes falsely reassure consumers. These products, which are far from harmless, may present a risk under certain conditions of use, be it nephrotoxic, hepatotoxic, or a cause of severe allergies [31]. In France, ANSES, the National Agency for Food, Environmental and Occupational Health and Safety provides some advice on its website. Consumers are asked to seek advice from a health professional to avoid prolonged, repeated, or multiple doses of plant-based food supplements, to respect the conditions of use, to be vigilant regarding products presented as miraculous, and to give preference to products sold through legal channels. It is also recommended that health professionals advising on and selling plant-based food supplements be trained on the safety and use of plants contained in those products. Ultimately, the ANSES delivered a new tool to support healthcare professionals, the first in Europe to the best of our knowledge. The Agency has analyzed and adapted the existing terms and restrictions for herbal medicinal products, as well as transposing them to food supplements containing these same plants. From this reflection, an appendix listing all the precautions for use, recommendations, and potential drug interactions relating to 118 medicinal plants used in food supplements was compiled. To disseminate this, an Excel table summarizing information for each plant was created and is accessible online. Primarily intended for doctors, pharmacists, and nutritionists, it aims to provide better support for consumers of food supplements. The list was last updated in June 2023 and is accessible from the following URL (https://www.anses.fr/fr/content/tableau-recapitulatif-plantes-complements-alimentaires, accessed on 4 July 2024) [32]. This food safety mission is currently under the supervision of the Ministry of Agriculture since January 2024.

This work follows former regulation on plant-based dietary supplements in the EU. In particular, a list of 1011 plants (other than fungi) authorized in food supplements and the conditions for their use is provided, along with the criteria of absence of toxicity highlighted [33,34].

The lack of disclosure of pharmaceuticals in food supplements circumvents clinician oversight of drug use, and in addition to side effects related to the specific pharmaceutical substance itself, risks also include potential and actual interactions with prescription drugs [10,35]. Another risk of nondisclosure of these APIs lays in the fact that when an adverse event occurs, it might not be properly attributed to the API that caused it and corrective therapy could be delayed. In addition, patients might simply assume they had an issue with one of the disclosed ingredients and move on to another product that again has the hidden API they adversely reacted to previously [7]. The alarming number of RASFF notifications on the illegal use of pharmaceuticals in food supplements clearly shows that the existing regulations do not efficiently prevent irresponsible manufacturers from falsifying their products [10]. In the US, in several instances, the FDA’s identification of an API in a dietary supplement resulted in voluntary withdrawal, product seizure, or legal action against the manufacturers, distributors, or sellers [7,36].

According to the EU General Food Law Regulation (EC) No 178/2002, food supplements are considered foodstuffs and consequently regulated as foods; therefore, they must not contain APIs. Responsibility for the safety of these products lies with the food business operator placing the product on the market [3]. Like the pharmacovigilance for medicines, the ANSES has implemented a food supplement surveillance system (nutrivigilance) in France, accessible from the URL https://www.nutrivigilance-anses.fr/nutri#, accessed on 4 July 2024.

Healthcare professionals are advised to ask their patients about their consumption of food supplements and to report any undesirable effects or adverse reactions they are aware of to the nutrivigilance system in order to help improving product knowledge and consumer safety. This initiative should be replicated globally. The European Food safety agency (EFSA), the RASFF, and the US FDA could lead this endeavor. It is clear that change in the legislation of food supplement will evolve from a real implication of all stakeholders, whether they are consumers’ associations, legislators, official controls laboratories, customs, clinicians, pharmacists, or academic researchers.

## 3. Materials and Methods

### 3.1. Chemicals and Reagents

For UHPLC-HRMS, solvents and reagents used in sample preparation and mobile phase were of Optima LC-MS-grade. Methanol (MeOH), water (H_2_O), ammonium formate, and formic acid were purchased from Fisher Scientific (Illkirch, France). Sibutramine hydrochloride CRM (≤100%, S-011-1 mL, 1.0 mg/mL in MeOH) and Diclofenac analytical standard (≥98.5%, 93484-100 mg) were purchased from Sigma-Aldrich (St. Quentin Fallavier, France). Working standard solutions were prepared in MeOH.

For HPLC-UV/MS analysis, quality grade MeOH, acetonitrile (ACN) and formic acid were obtained from Carlo Erba (Val de Reuil, France). Ultrapure water (18.2 MW.cm) was obtained from Elga and Purelab Classic (VeoliaWater, Antony, France). Pure diclofenac was isolated from the dichloromethane (CH_2_Cl_2_) extract of diclofenac Zentiva gel (containing 1.16% (*m*/*m*) of diclofenac diethylammonium salt, Zentiva France, Paris, France). The gel (5.29 g) was dispersed in water (50 mL) and the pH adjusted to pH 2 with few drops of aqueous concentrated hydrochloric acid (HCl). The mixture was sonicated for 30 min, filtered through Whatman N°1 filter paper, and the resulting solution extracted twice with 50 mL of CH_2_Cl_2_. Diclofenac free acid was obtained as a white solid after solvent evaporation (300 mg).

For NMR analysis, deuterated solvent (CDCl_3_ 99.8% D and DMSO-*d6* 99.8% D) was obtained from Eurisotop (Saint-Aubin, France). Dexamethasone 21-acetate (≥99%, D1881-100 mg, batch 1003506634) and Betamethasone 21-acetate CRS (≤100%, code B1030000, batch 4.0, lot 01576) were purchased from Sigma-Aldrich (St Louis, MO, USA) and European Pharmacopoeia (Strasbourg, France), respectively.

For XRPD analysis, white powder of Piroxicam (≥97%, P0847, batch 044K1161) and diclofenac sodium (≤100%, 287840-1g, lot 349279) were sourced from Sigma-Aldrich (St Louis, MO, USA) and Millipore (Darmstadt, Germany), respectively. White powders of dexamethasone 21-acetate and betamethasone 21-acetate were the same used for NMR analysis.

### 3.2. Test Materials

Sample materials include two plant-based food supplements, namely Meng yululu and Castanha da India Indiana. The capsules of the slimming product, Meng yululu, were obtained through Paris Poison Center, from an intoxicated patient gone through hospitalization care unit. Four batches of Castanha da India Indiana were purchased online in December 2019. Castanha da India Indiana products, very popular in Brazil, are advertised to be anti-inflammatory, to facilitate blood circulation, and to present anti-hemorrhoidal properties. They are supplied in capsule form in plastic bottles containing 15 units and will be referred in the text as samples B1, B2, V1 and V2, respectively.

**Figure 7:**
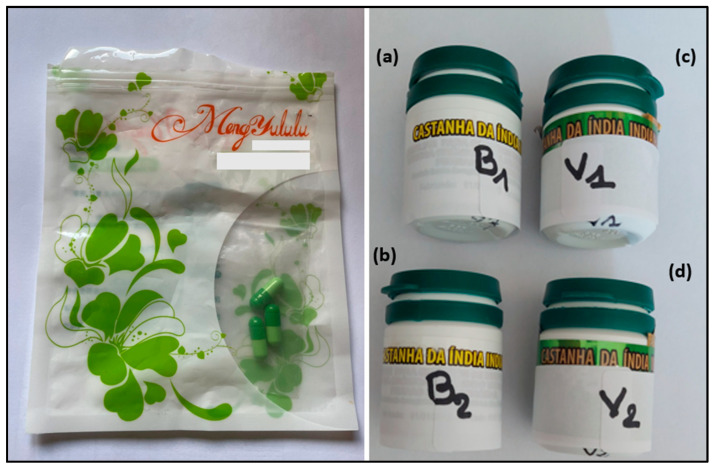
Samples investigated in the study, including plant-based food supplements Meng yululu (**left**) and Castanha da India Indiana (**right**). Meng Yululu, Herbal slimming product, capsules of food supplement supposed to be of plant origin and obtained from a patient after admission to hospital. Castanha da India Indiana, food supplements supposed to be of plant origin; (**a**) sample B1, (**b**) sample B2, (**c**) sample V1, and (**d**) sample V2.

The packaging for the specimens are presented in Figure 7.

### 3.3. Physico-Chemical Analyses

#### 3.3.1. UHPLC-HRMS/MS Q-Exactive™ Analysis

Meng yululu and Castanha da India Indiana samples (lots B1, B2, V1 and V2) were extracted following a procedure adapted from Qingfang Meng et al. [37]. Briefly, 5 mL of MeOH/H_2_O (95/5 *v*/*v*) containing 0.005% of formic acid was added to 50 mg of test material (powder content of the capsules). The sample was subsequently vortexed, sonicated for 15 min, and centrifuged (8000× *g*, 10 min, 4 °C). The supernatant was collected and evaporated to dryness using a SpeedVac concentrator. The dry extract was reconstituted with 500 μL of a solution of 20% aqueous MeOH (initial conditions of the chromatographic elution gradient). The final extract was spin-filtered prior to UHPLC–HRMS analysis.

A Dionex™ UltiMate™ 3000 UHPLC system (Dionex, Sunnyvale, CA, USA) coupled to a Thermo Scientific Q Exactive™ HRMS (Thermo Fisher Scientific, Bremen, Germany) equipped with a heated electrospray (HESI II) source was used for sample analysis and accurate mass measurements. Reverse-phase chromatography conditions combined with electrospray ionization were applied.

The UHPLC system was equipped with a Hypersil Gold C18 column (1.9 μm, 100 mm × 2.1 mm) with the corresponding guard column. The analytical column and the autosampler were maintained at 40 °C and 4 °C, respectively.

The mobile phase consisted of H_2_O with 0.1% formic acid and 5 mM ammonium formate (pH 3.13) in channel A, and MeOH containing 0.1% Formic acid in channel B. The system was programed to perform gradient elution from 20% to 50% B over a 2 min period, 50% to 90% B for 6 min, 90% to 98% B for 3 min, held at 98% B for 5 min, returned to initial conditions for over 4 min, and then held at these conditions for a further 3 min. The flow rate was 0.4 mL/min and the injection volume was 5 μL.

The mass spectrometer operated in positive (HESI^+^) ionization mode with the source parameters as follows: spray voltage 3.5 kV, capillary temperature 320 °C, sheath gas flow rate 40 a.u. (arbitrary units), auxiliary gas flow rate 15 a.u., sweep gas flow rate 0 a.u., S lens RF level 50, and heater temperature 400 °C.

Data acquisition was carried out in Full scan MS and Data-dependent MS/MS.

Full scan mass spectra were acquired from 60 to 900 *m*/*z* using resolution 70,000 Full Width at Half Maximum (FWHM) with automatic gain control (AGC) target of 1 × 10^6^ ions and a maximum ion injection time (IT) of 120 ms.

MS/MS experiments were performed in data-dependent MS/MS (ddMS2) ‘Top5’ mode using the following settings: resolution 17,500 FWHM with automatic gain control (AGC) target of 2 × 10^5^ ions, a maximum IT of 50 ms, an isolation window of 0.4 *m*/*z*, and a normalized collision energy (NCE) of 30 ± 50%.

Xcalibur 3.1 software, Freestyle 1.8 SP1, and mzVault 2.3 (Thermo Fisher Scientific, San Jose, CA, USA) were used for instrument control, data visualization, and data processing.

#### 3.3.2. HPLC-UV/MS Analysis

Five capsules of each Sample B1, B2, V1, and V2 of Castanha da India Indiana, respectively, were randomly selected, their content accurately weighed (the filling weight of the five capsules ranged from 2.289 to 2.794 g), and subjected to sonication (Branson ultrasonic bath 2510, Danbury, CT, USA) for 1 h in a flask with 25 mL of CH_2_Cl_2_. Crude extracts were filtered through Whatman N°1 filter paper and the resulting solution was evaporated to dryness in a rotary evaporator (Büchi Rotavapor R-200, Flawil, Switzerland). Test solutions and diclofenac reference solution were prepared by weighing 5 mg of the dried crude CH_2_Cl_2_ extracts and 15 mg of diclofenac free acid, respectively, before dissolving them in 3 mL of MeOH (LC-MS grade). All solutions were filtered through 0.2 µm nylon syringe filters prior to injection. During preliminary work, diclofenac was extracted from pharmaceutical preparation, and subsequently an analytical standard of diclofenac was purchased.

The instrument consisted of HPLC-DAD-MS ThermoScientific Dionex U3000 (Thermo-Dionex, Les Ulis, France) coupled to a quadrupole mass spectrometer (Surveyor MSQ plus System, Thermo-Dionex, Les Ulis, France). The analytical column was a Zorbax SB C18 (3 µm, 150 Å, 100 mm × 2.1 mm) maintained at 25 °C.

The mobile phase consisted of H_2_O with 1% formic acid in channel A and ACN in channel B. The system was programed to perform gradient elution from 90% to 70% A for over 20 min, 70% to 30% A for 10 min, held at 30% A for 25 min, and returned to initial conditions for over 5 min. The pump flow rate was 0.6 mL/min and the injection volume was 20 µL.

The MS was operated in the negative ionization mode with the following conditions: Ion spray voltage 3 kV, curtain gas 50 psi, Quadrupole energy 70 V, cone voltage 50 V, desolvation temperature 500 °C, and ion energy 0.8 V. Mass spectra were acquired within the range of 100–1000 *m*/*z*. Chromeleon^®^, version 6.8 software provided by Thermo Scientific Dionex (Les Ulis, France) was used for data processing. Detection at definite wavelength 254 nm was used to record the chromatograms.

#### 3.3.3. NMR Analysis

NMR spectra of standards and selected extracts of the dietary supplements (i.e., samples B1, B2 of Castanha da India Indiana, and sample of Meng yululu) were recorded on Bruker AC300 (300 MHz) and Bruker Avance III HD (400 MHz) instruments and processed with TopSpin Bruker 4.4.0 software. ^1^H NMR spectra were calibrated using the residual peaks of solvent as internal reference (DMSO-*d6*: δ_H_ 2.50 ppm). Chemical shifts (*δ*) were given in parts per million (ppm) and coupling constants (*J*) were given in hertz (Hz). Signals were described as singlet (s), doublet (d), doublet of doublet (dd), triplet (t), etc. Samples were prepared by accurately weighing approximately 10 mg of standard or extract, respectively, and dissolved in 750 µL of DMSO-*d6*. The sample preparation has already been described in Section 3.3.2.

#### 3.3.4. X-Ray Powder Diffraction (XRPD) Analysis

XRPD measurements were performed with a Malvern Panalytical Aeris (Worcestershire, England) at room temperature with an incident wavelength of λCu = 1.54 Å. The data were recorded from 5° to 60° in 2Theta with a 0.02° step. Once XRPD data was acquired, the positions and intensities of the observed peaks were compared with those calculated from the structures found in the databases or literature, and quickly checked whether our sample has the correct crystalline phase, is free of impurities, etc.

Diclofenac sodium reference standard was used as such. Diclofenac sodium can crystallize in many different forms depending on the manufacturing and formulation conditions. Three hydrated forms of diclofenac can be found in the literature [38,39,40]. One crystalline structure of the anhydrous diclofenac sodium has been determined up to now [41] and the commercial diclofenac sodium powder corresponds to this anhydrous crystalline form (Supplemental Figure S12a).

Piroxicam reference standard was used as such. Piroxicam presents crystallographic polymorphism, and the crystal structure of 5 forms of pure anhydrous piroxicam (I, IIa1, IIa2, III and IV) can be found in the Cambridge Structural Database (CSD). X-ray powder diffraction (XRPD) data were recorded and the polymorph I was identified (Figure S12b).

Dexamethasone 21-acetate reference standard was used as such. Two polymorphs of the monohydrated form have been reported but no crystalline structure can be found for the anhydrous form [42]. XRPD pattern was recorded and compared to the calculated pattern of dexamethasone 21-acetate monohydrate form I (Figure S12c).

Betamethasone 21-acetate was used as such. Unfortunately, no crystalline structure of betamethasone acetate has been found in the literature. The XRPD pattern is presented in Figure S12d.

Powder content of Meng yululu and Castanha da India Indiana (lots B1, B2, V1, and V2) commercial herbal food supplement capsules were screened without any preparation. Samples from reference standards and tested materials were put on a metallic sample holder and placed on the X-ray diffractometer.

## 4. Conclusions

Adulteration of food supplements by the illegal addition of pharmaceuticals is still a reality. The analytical strategy reported here employed an integrated physico-chemical approach and performed adequately. XRPD, LC-UV/MS, LC-HRMS/MS, and NMR indicated food supplement adulteration by diclofenac, piroxicam, dexamethasone 21-acetate, and sibutramine. The surprising detection of those APIs gave a strong indication that they were added intentionally to deceive consumers about the miraculous benefits of food supplements sold. These food supplements can be categorized as “medicines in disguise”, which represent a major public health challenge. Health concerns associated with the consumption of “medicines in disguise” appeal to establishing a coherent food policy and underscore the importance for consumers to purchase food supplements in the legal circuit. Vigilance systems only exist in the context of product legality (e.g., ANSES nutrivilance system, RASFF, EFSA, etc.) and hence the interest of the FalsiMedTrack project, which can also pinpoint products adulteration outside the legal circuit.

Current challenges in the field remain scientific and regulatory, as well as public awareness in safeguarding their health and safety.

## Figures and Tables

**Table 1:** Characteristics of active pharmaceuticals identified in plant-based food supplements and raw materials.

Plant-Based Food Supplements and Raw Materials	ID of Detected Pharmaceuticals	Molecular Formula	Retention Time (min)	Detected *m*/*z*	Mass Accuracy (Δ ppm)	Ions	HRMS/MS Fragment Ions	Analytical Method
Castanha da India Indiana capsules—samples V1 and V2	Diclofenac	C_14_H_10_Cl_2_NO_2_	26.70 7.52	293.8 296.02429	1.11	[M − H]^−^ [M + H]^+^	278.0136, 250.0186 and 215.0496	HPLC-UV/MS UHPLC-HRMS/MS
Castanha da India Indiana capsule—samples B1 and B2	Piroxicam	C_15_H_13_N_3_O_4_S	20.11 4.77	329.9 332.06973	0.66	[M − H]^−^ [M + H]^+^	164.0819, 121.0399 and 95.0609	HPLC-UV/MS UHPLC-HRMS/MS ^1^H NMR XRPD
Castanha da India Indiana capsule—samples B1 and B2	Dexamethasone 21-acetate	C_24_H_31_FO_6_	24.06 6.37	433 435.21777	0.07	[M − H]^−^ [M + H]^+^	415.2132, 397.2006, 379.1890, 355.1905, 337.1796, 319.1692 and 309.1890	HPLC-UV/MS UHPLC-HRMS/MS ^1^H NMR
Meng yululu capsules	Sibutramine	C_17_H_26_ClN	5.47	280.18286	0.75	[M + H]^+^	139.0309 125.0155	UHPLC-HRMS/MS

## Data Availability

Data are contained within the article and Supplementary Materials.

## Supplementary Materials

The following supporting information can be downloaded at: https://www.mdpi.com/article/10.3390/molecules30061372/s1, Figure S1: X-ray powder diffraction patterns of the food supplements analyzed in the study; Figure S2: Reverse phase HPLC-UV chromatograms of MeOH reconstituted CH_2_Cl_2_ extract from Castanha India Indiana commercial herbal food supplement samples B1, B2, V1, and V2, and diclofenac standard (isolated from diclofenac Zentiva gel) (from top to bottom panel); Figure S3: Reverse phase HPLC-UV chromatograms, UV/Visible spectra, and ESI^−^ MS spectra of major peaks in Sample V1 and Sample V2, identified as diclofenac; Figure S4: UHPLC-HESI^+^ Q Exactive Orbitrap HRMS/MS chromatograms of plant-based food supplement samples V1, V2, diclofenac analytical standard; Figure S5: HPLC-UV chromatogram, UV/Visible spectra, and ESI^−^ MS spectra of major peaks in Sample B1 on the new HPLC gradient used; Figure S6: UHPLC-HESI^+^ Q Exactive Orbitrap HRMS TIC chromatogram of Sample B2, full MS spectrum, and ddMS2 fragmentation spectrum of Piroxicam; Figure S7: UHPLC-HESI^+^ Q Exactive Orbitrap HRMS TIC chromatogram of Sample B2, full MS spectrum, and ddMS2 fragmentation spectrum of Dexamethasone/Betamethasone acetate; Figure S8: Fragmentation pattern of Dexamethasone acetate in mzCloud compared to Sample B2; Figure S9: 1H NMR spectrum in DMSO-*d6* of the crude CH_2_Cl_2_ extract of Sample B2 with NMR signal integration for piroxicam; Figure S10: ^1^H NMR spectrum in DMSO-*d6* of the crude CH_2_Cl_2_ extract of Sample B2 with NMR signal integration of non-overlapped signals of dexamethasone 21-acetate; Figure S11: Integrated ^1^H NMR spectrum of dexamethasone 21-acetate in DMSO-*d6*; Figure S12: Overlay of the experimental XRPD pattern of the commercial sodium diclofenac, piroxicam, dexamethasone acetate, bethamethasone acetate, and their calculated patterns.

## Abbreviations

Food supplements (FS), active pharmaceutical ingredient (API), ultra-high performance liquid chromatography (UHPLC), high-resolution mass spectrometry (HRMS), nuclear magnetic resonance (NMR), X-ray powder diffraction (XRPD), diffusion-ordered spectroscopy (DOSY), Correlated Spectroscopy (COSY), coulometric electrode array (CEA), time-of-flight mass spectrometry (TOF-MS), infrared spectroscopy (IR), ultra violet (UV), total ion current (TIC), quadrupole (Q), European Union (EU), compound annual growth rate (CAGR), Phosphodiesterase-5 inhibitors (PDE-5i), European Directorate for the Quality of Medicines and Healthcare (EDQM), Official Medicines Control Laboratories (OMCL), General European OMCL Network (GEON), Rapid Alert System for Food and Feed (RASFF), methanol (MeOH), dichloromethane (CH_2_Cl_2_), Acetonitrile (ACN), calcium carbonate (CaCO_3_), non-steroidal anti-inflammatory drug (NSAID), cyclooxygenase (COX), Acute Respiratory Disease Syndrome (ARDS), dexamethasone 21–acetate (DA), betamethasone 21–acetate (BA), dimethylsulfoxyde (DMSO), Inorganic Crystal Structure Database (ICSD), water (H_2_O), hydrochloric acid (HCl), deuterated chloroform (CDCl_3_), Agence nationale de sécurité sanitaire de l’alimentation, de l’environnement et du travail (ANSES), European Food safety agency (EFSA), FDA (food and drug agency).
