# Supplementary material for: Cross-Analytical Strategies to Tackle “Medicines in Disguise” Presented as Food Supplements, a New Threat for Human Health
Source: Molecules. 2025 Mar 19;30(6):1372. doi: 10.3390/molecules30061372 (PMC11944288; doi:10.3390/molecules30061372)
Supplement: Supplementary file 1 [file molecules-30-01372-s001.zip › molecules-3486886-supplementary.pdf]

Cross-analytical strategies to tackle "medicines in disguise" presented as food supplements; a new threat for human health

Judith Nzoughet Kouassi [1*], Chouaha Bouzidi [1], Béatrice Nicolai [1,2], Farah Ben Jamaa [1], Annabelle Dugay [1], Jérôme Langrand [3], Dominique Vodovar [3], Pascal Houzé [4], Laurence Labat [4], Bruno Mégarbane [5], Cinzia Bocca [6], Pascal Reynier [6], Nicolas Guiblin [2], Sylvie Michel [1], Xavier Cachet [1]

[1] UFR de Pharmacie, Faculté de Santé, Université Paris Cité, CNRS, Cibles Thérapeutiques et conception de médicaments - UMR 8038, Paris F-75006, France
[2] Laboratoire SPMS, Centrale Supelec, Université Paris Saclay, Plateau de Moulon 3 rue Joliot-Curie, F-91192 Gif-sur-Yvette Cedex, France
[3] Centre antipoison et de toxicovigilance de Paris, Hôpital Fernand-Widal APHP, Inserm UMR-S 1144, Paris, France
[4] Laboratoire de Toxicologie, Hôpital Lariboisière APHP, Inserm UMR-S 1144, Paris, France
[5] Réanimation Médicale et Toxicologique, Hôpital Lariboisière APHP, Inserm UMR-S 1144, Paris, France
[6] Faculté de santé, Institut MITOVASC, UMR CNRS 6015, INSERM U1083, Université d'Angers, Angers, France

* Correspondence: xavier.cachet@u-paris.fr

Supplementary Materials

Table of Contents

Supplemental Figure S1..Page S-2
Supplemental Figure S2..Page S-3
Supplemental Figure S3..Page S-4
Supplemental Figure S4..Page S-5
Supplemental Figure S5..Page S-6
Supplemental Figure S6..Page S-7
Supplemental Figure S7..Page S-8
Supplemental Figure S8..Page S-9
Supplemental Figure S9..Page S-10
Supplemental Figure S10..Page S-11
Supplemental Figure S11..Page S-11
Supplemental Figure S12..Page S-12

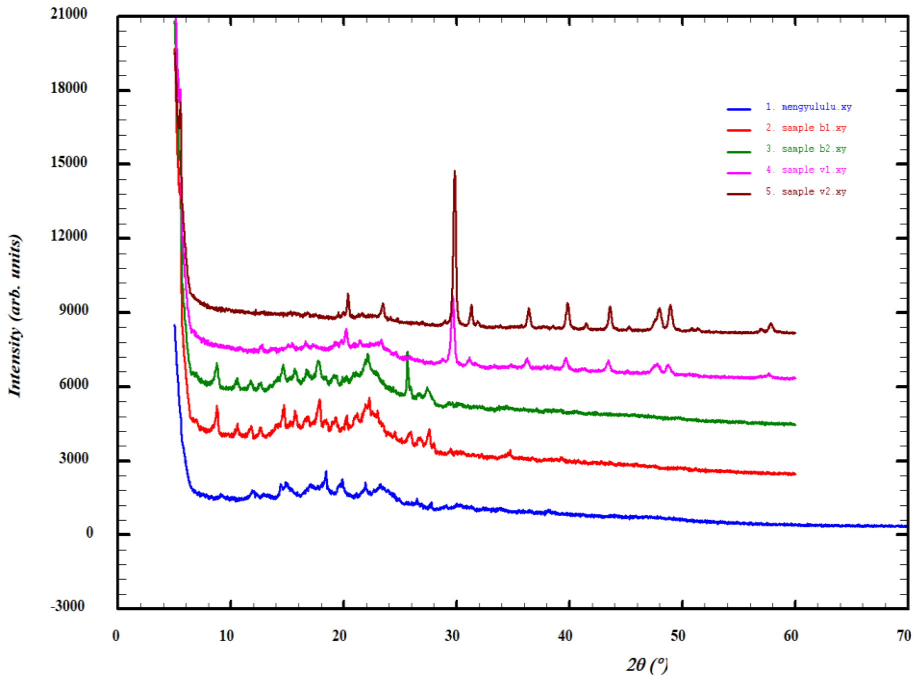

Figure S1: X-ray powder diffraction patterns of the food supplements analysed, i.e., Meng yululu (in blue), Castanha da India Indiana sample B1 (in red), Castanha da India Indiana sample B2 (in green), Castanha da India Indiana sample V1 (in pink), and Castanha da India Indiana sample V2 (in brown).

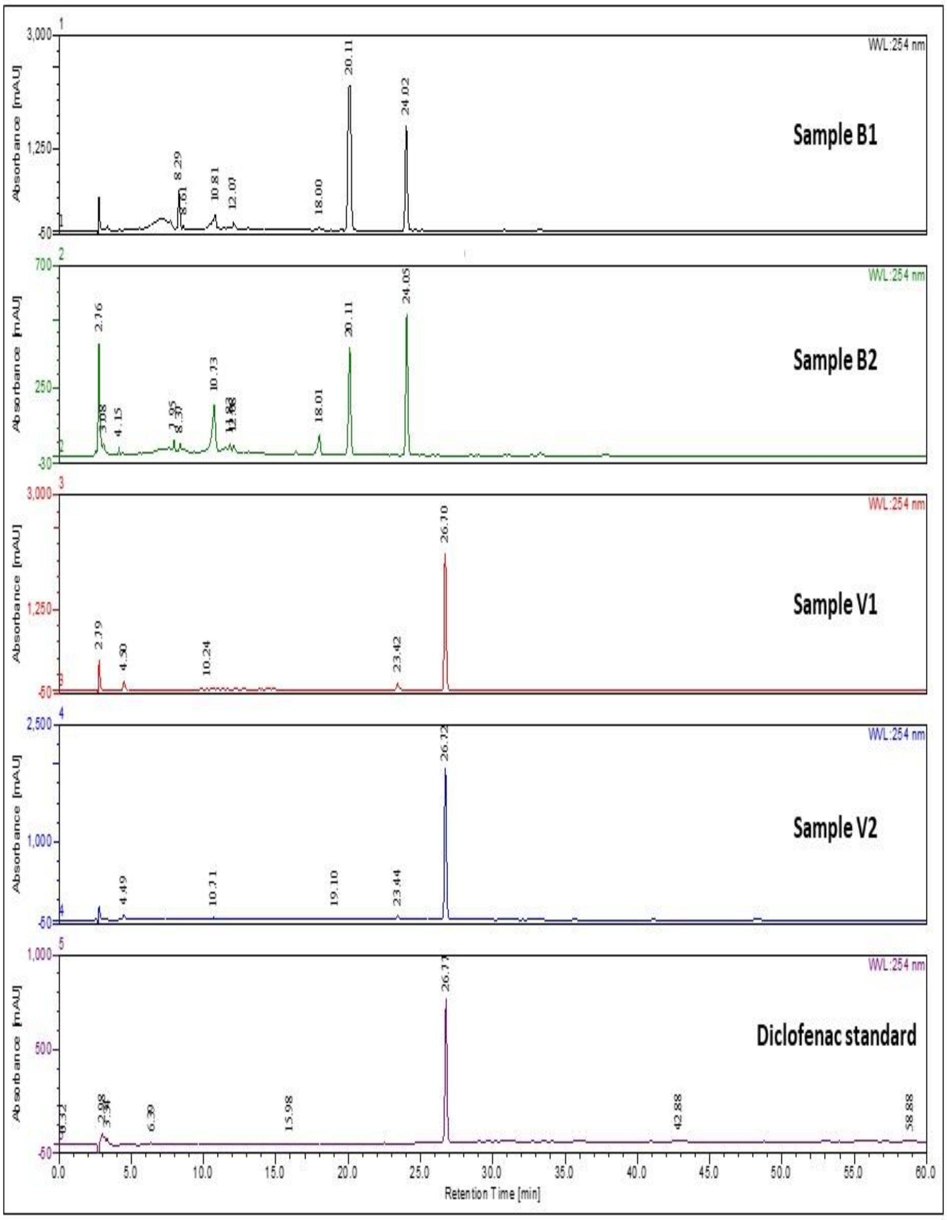

Figure S2. Reverse phase HPLC-UV chromatograms of MeOH reconstituted CH_2Cl_2 extract from Castanha India Indiana commercial herbal food supplement samples B1, B2, V1, V2, and diclofenac standard (isolated from diclofenac Zentiva gel) (from top to bottom panel).

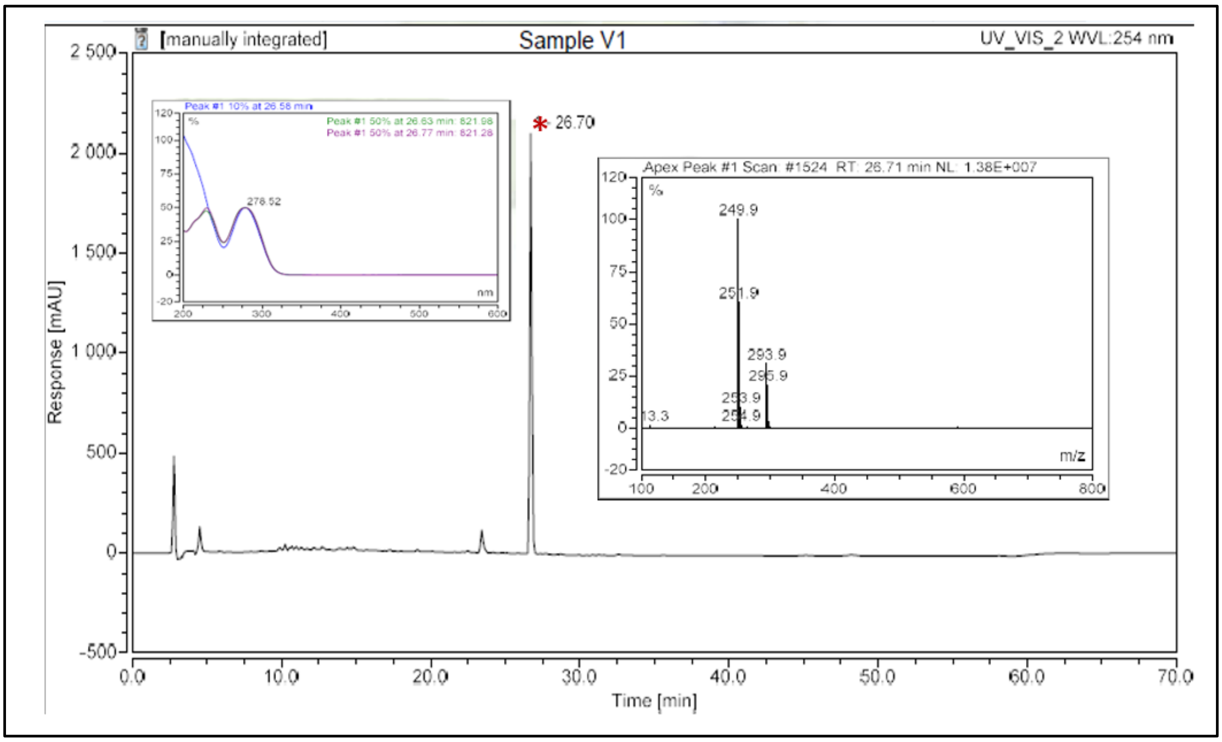

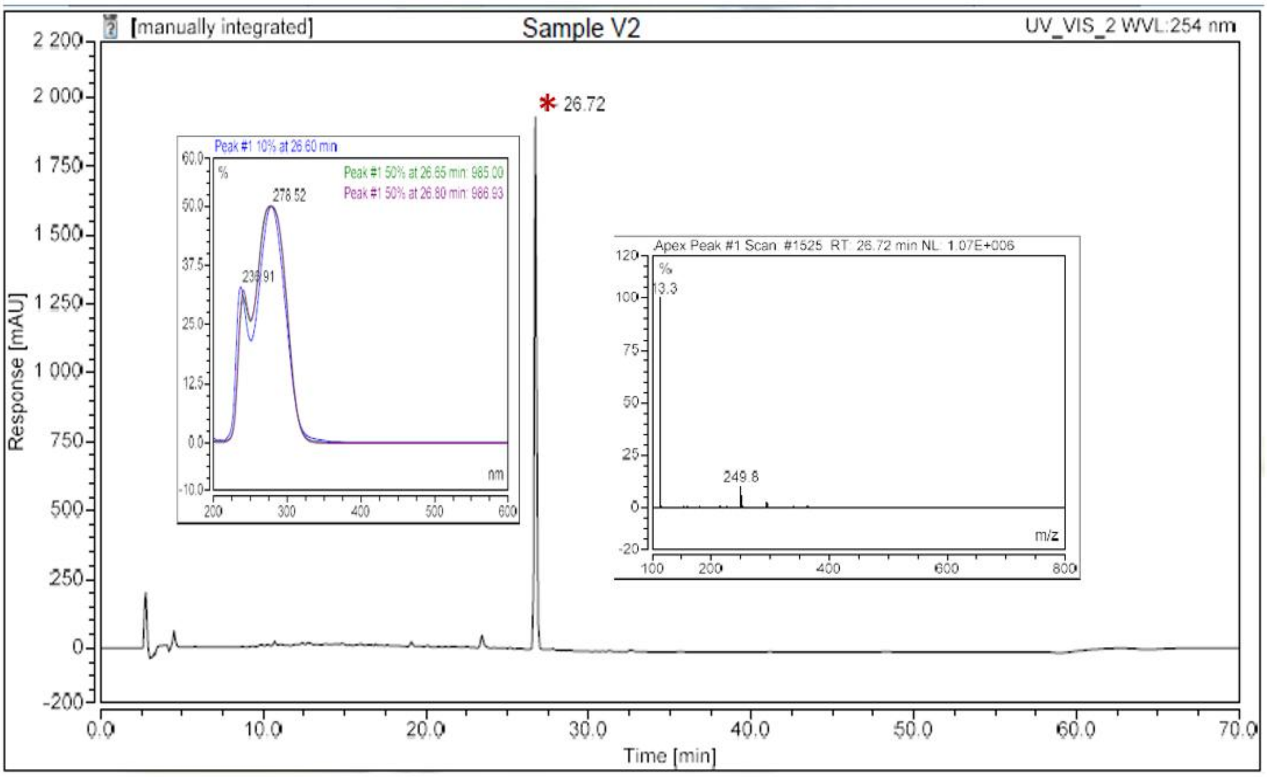

Figure S3: Reverse phase HPLC-UV chromatograms, UV/Visible spectra and ESI⁻ MS spectra of major peaks in sample V1 (top panel) and sample V2 (bottom panel), identified as Diclofenac

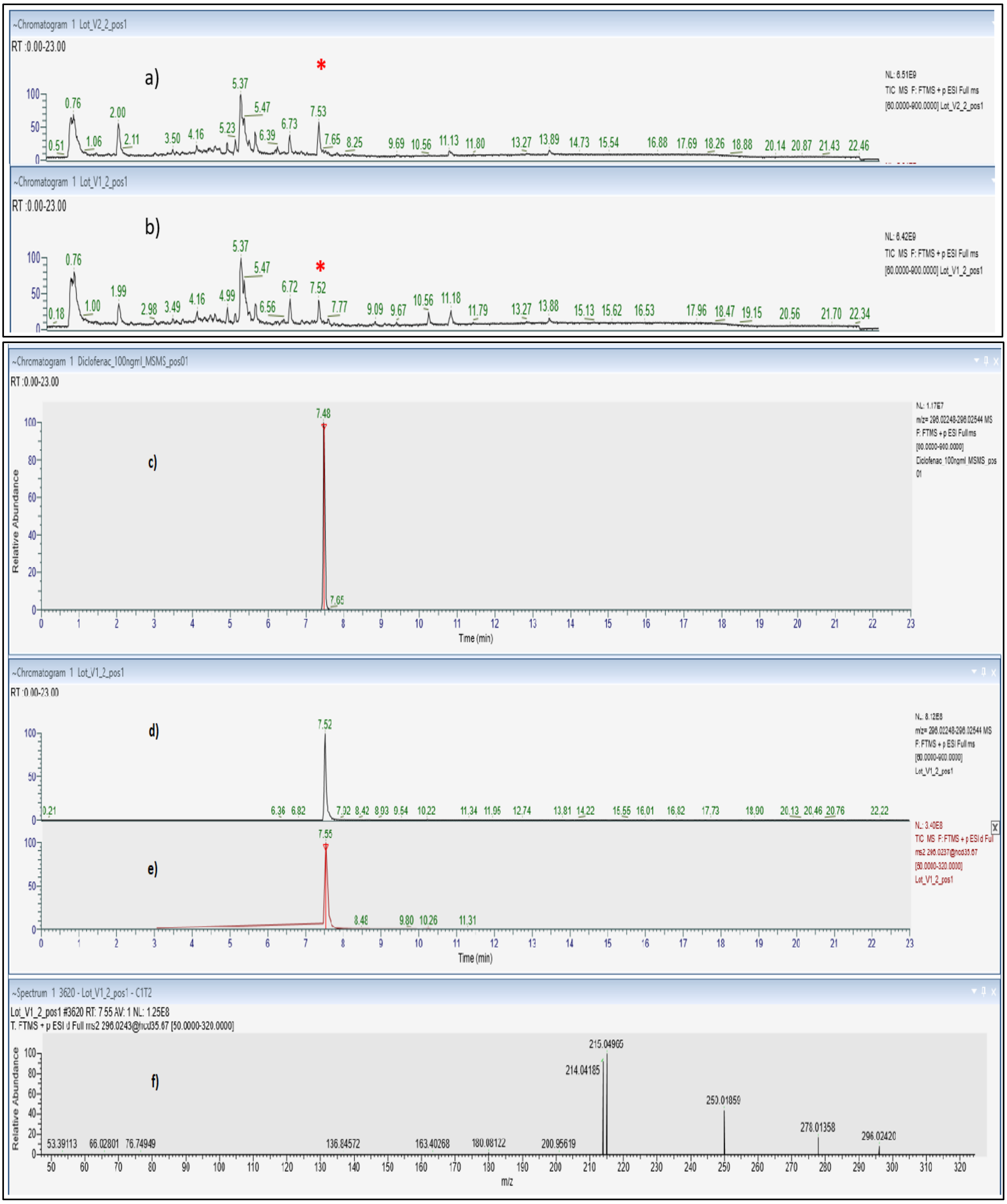

Figure S4: UHPLC-HESI+ Q Exactive Orbitrap HRMS chromatograms of plant-based food supplement samples V2 (a), V1 (b), diclofenac analytical standard (c), diclofenac base peak in sample V1, ddMS2 peak (d), and fragmentation spectrum (e) of diclofenac detected in sample V1.

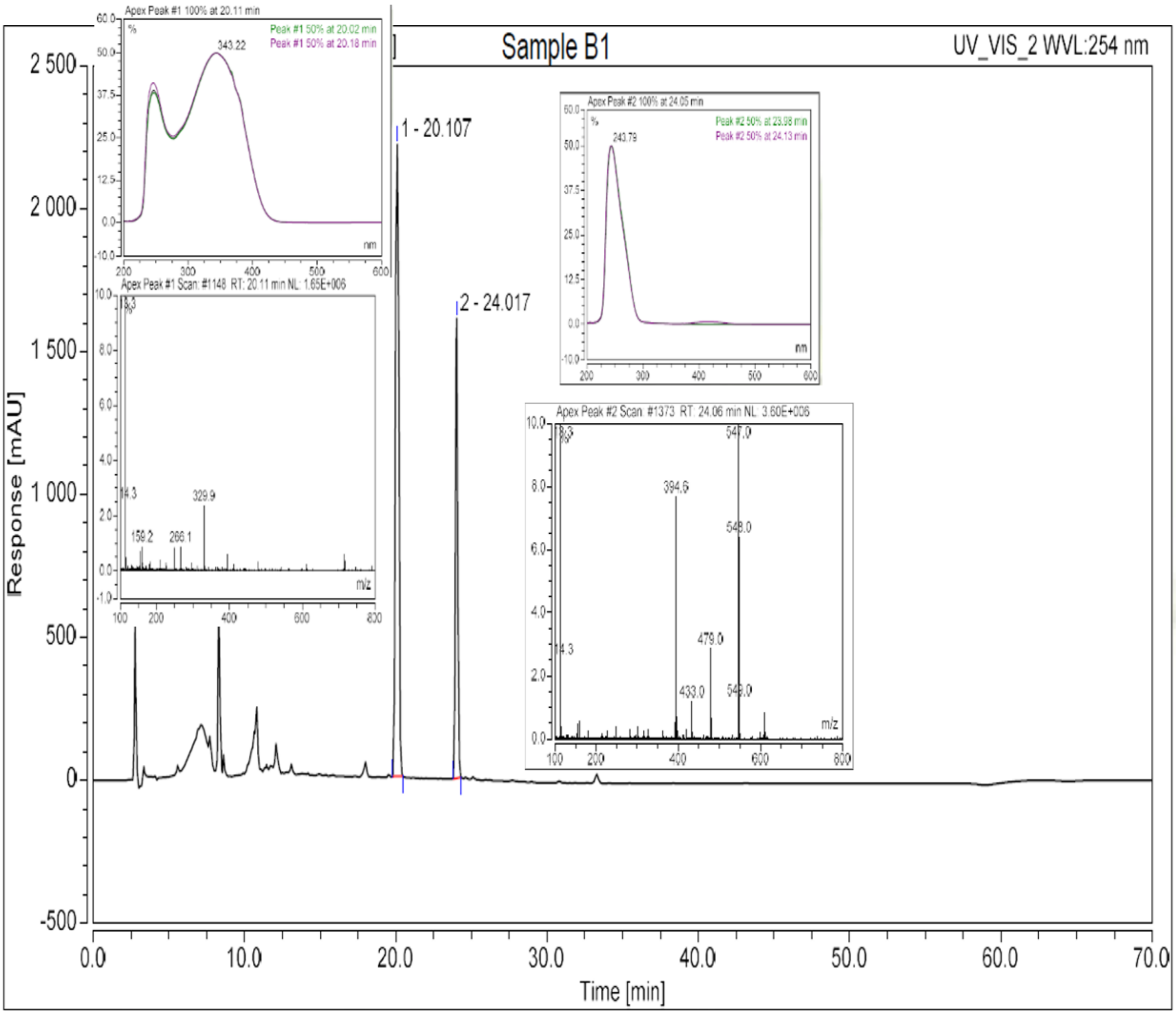

Figure S5: HPLC-UV chromatogram, UV/Visible spectra, ESI$^-$ MS spectra of major peaks in sample B1, eluting at 20 min and 24 min with $[M-H]^-$ adducts detected at *m/z* 329.9 and 433, respectively.

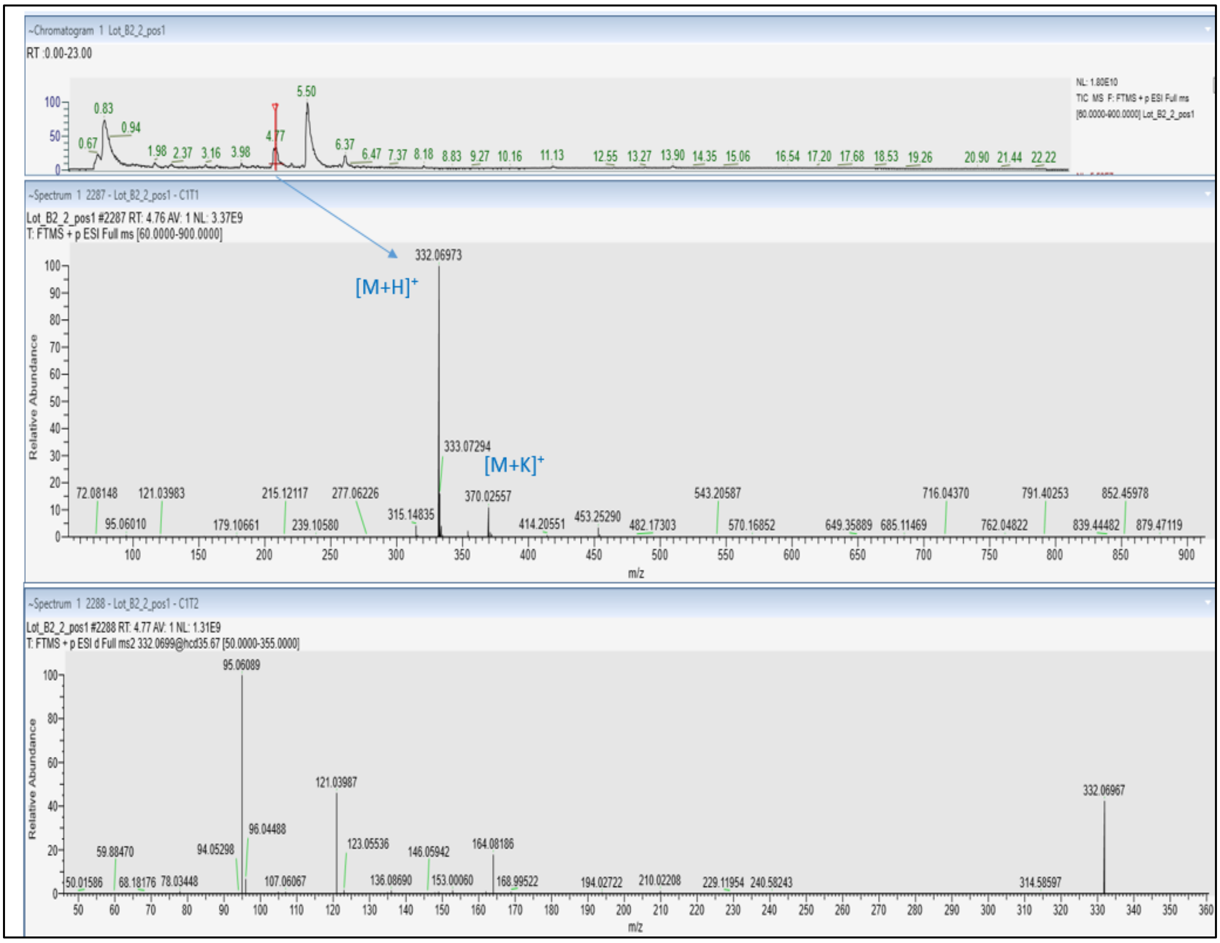

Figure S6: UHPLC-HESI+ Q Exactive Orbitrap HRMS TIC chromatogram of sample B2, full MS spectrum and ddMS2 fragmentation spectrum of Piroxicam detected at 4.77 min (top to bottom panel)

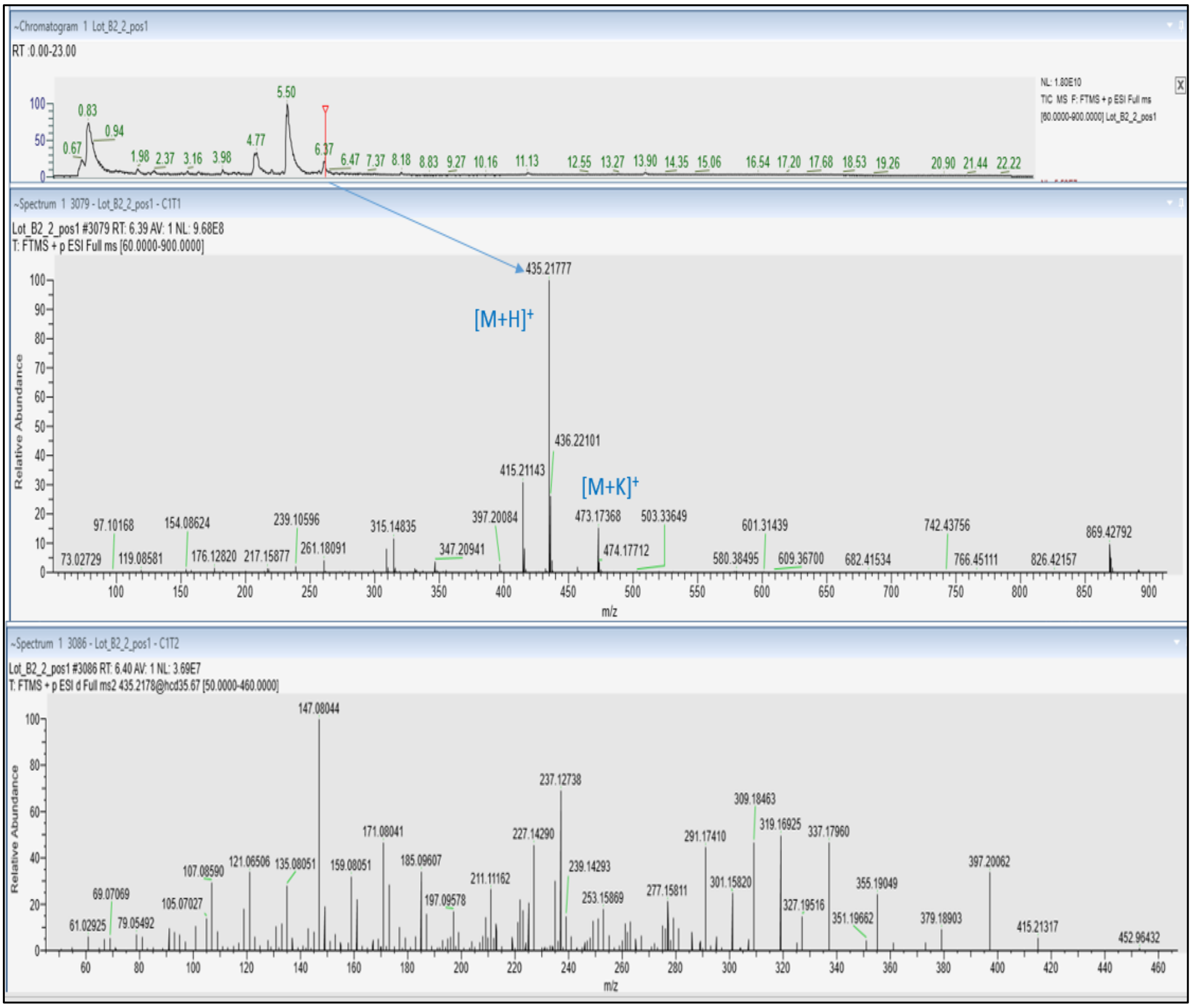

Figure S7: UHPLC-HESI+ Q Exactive Orbitrap HRMS TIC chromatogram of sample B2, full MS spectrum and ddMS2 fragmentation spectrum of dexamethasone/ betamethasone 21-acetate (DA and BA), flunisolide, or triamcinolone acetonide ($C_{24}H_{31}FO_6$) detected at 6.37 min (top to bottom panel).

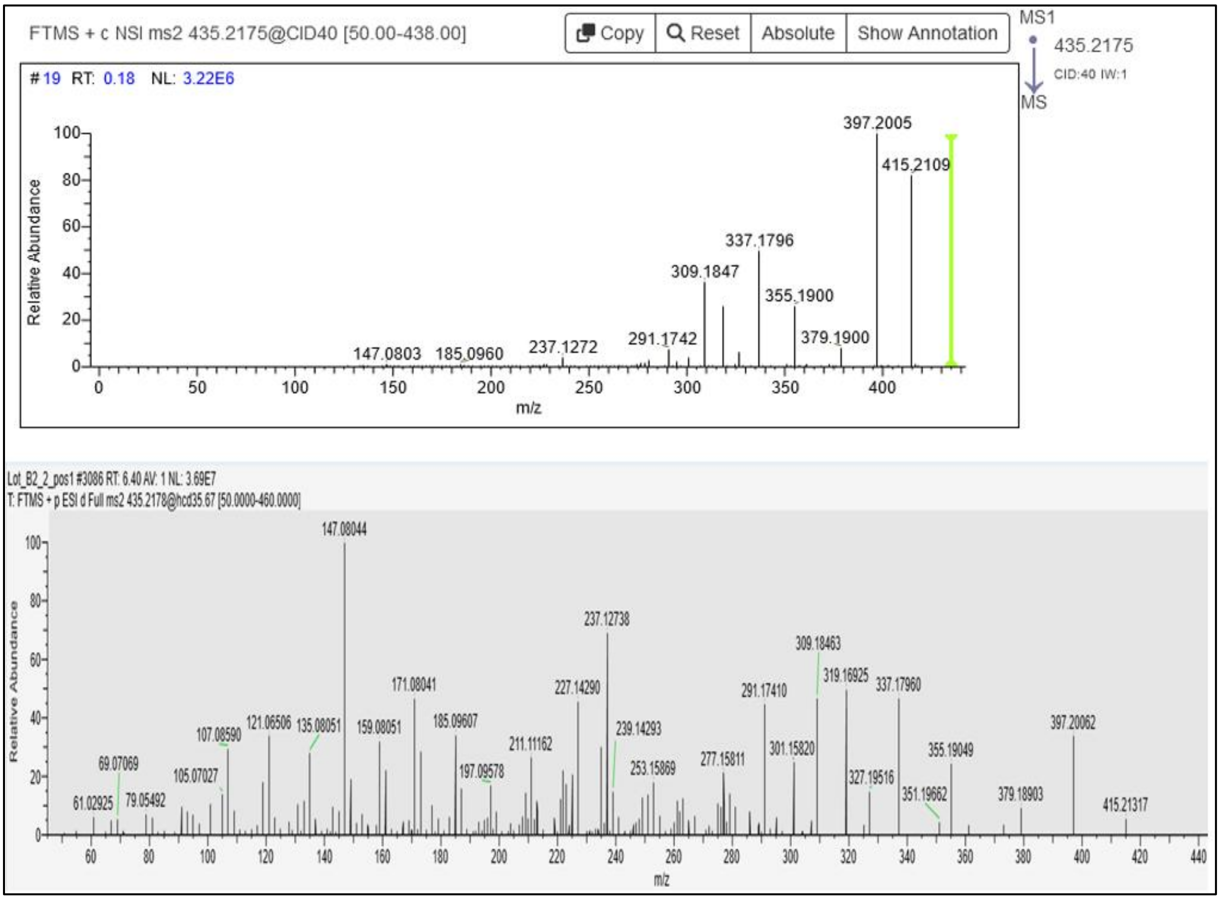

Figure S8: Fragmentation pattern of Dexamethasone 21-acetate in mzCloud (high resolution mass spectral database from Thermo Fisher scientific, top panel) compared to sample B2 (bottom panel).

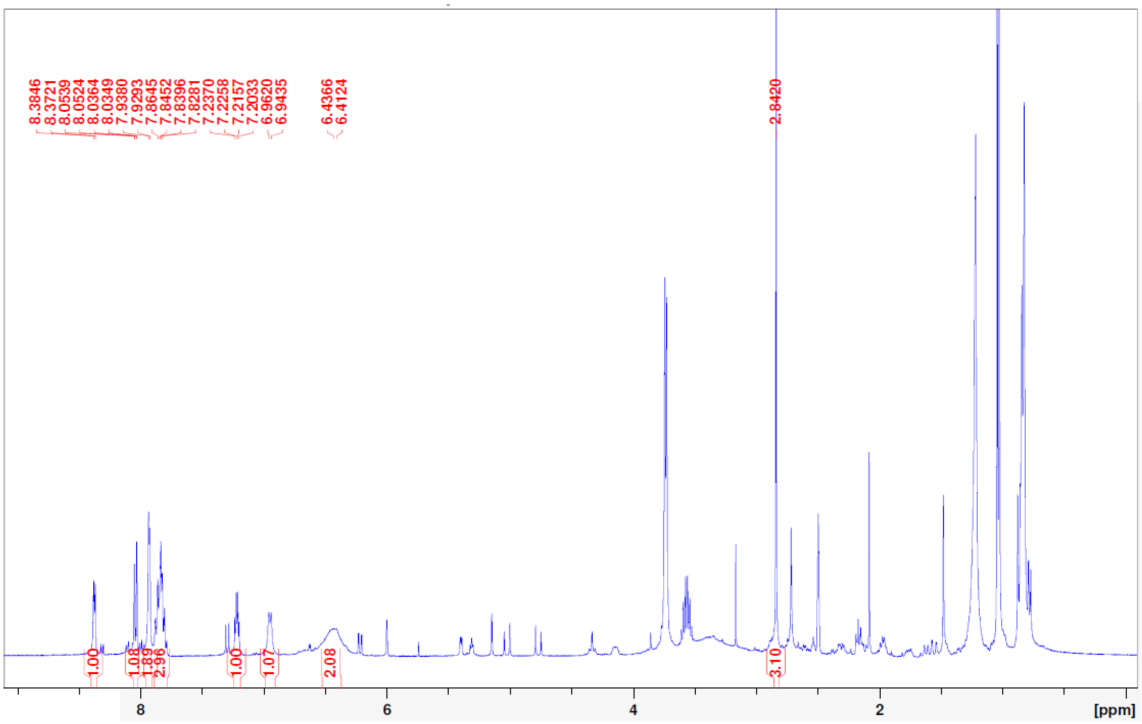

Figure S9: ^{1}H NMR spectrum in DMSO-*d6* of the crude CH_2Cl_2 extract of sample B2 with NMR signal integration for piroxicam.

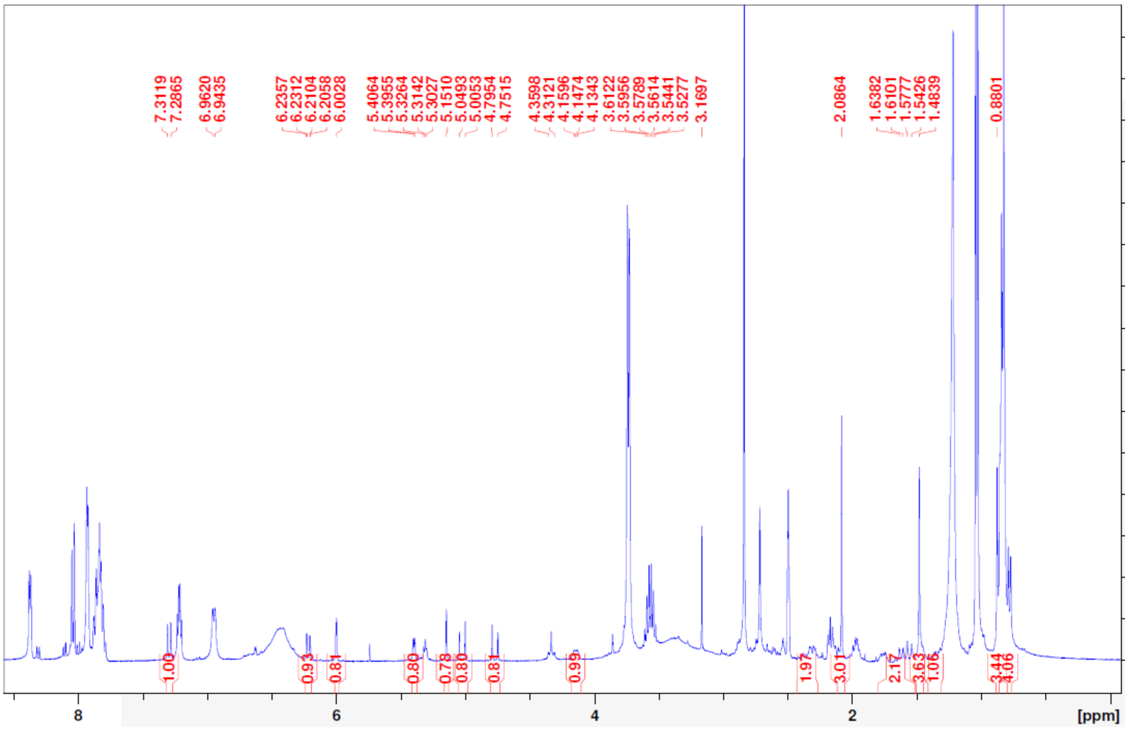

Figure S10: ^{1}H NMR spectrum in DMSO-*d6* of the crude CH_2Cl_2 extract of sample B2 with NMR signal integration of non-overlapped signals of dexamethasone 21-acetate.

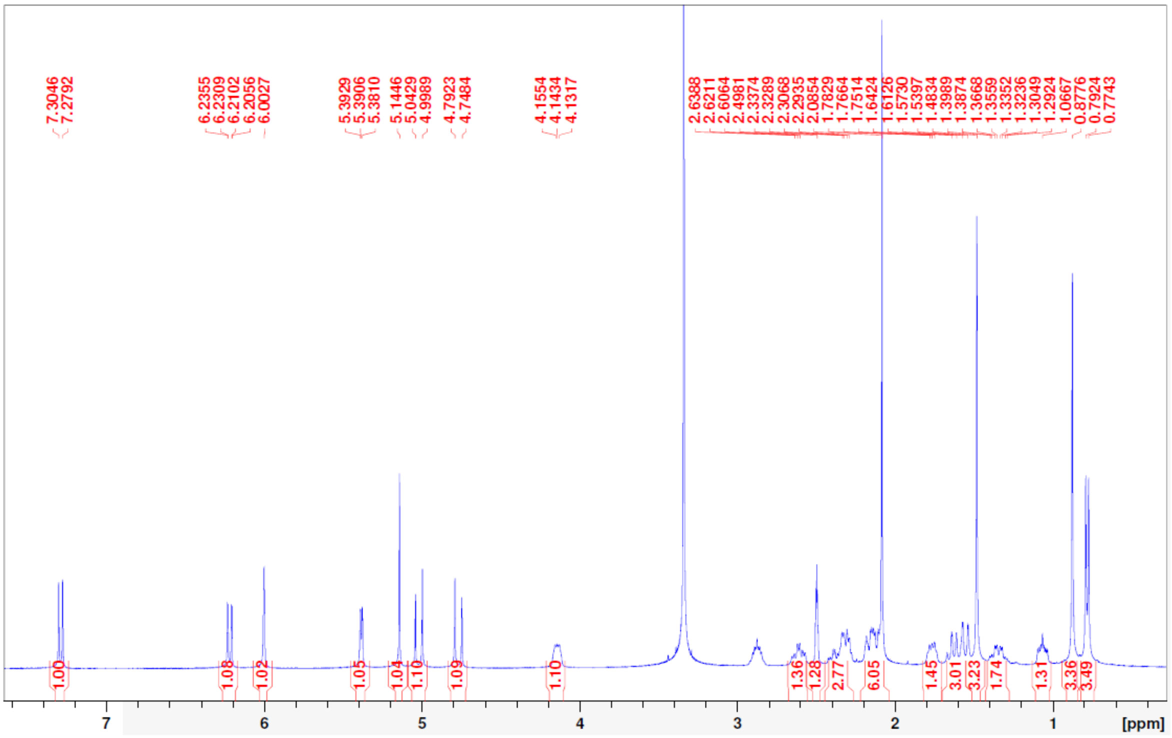

Figure S11: Integrated ^{1}H NMR spectrum of dexamethasone 21-acetate in DMSO-*d6*.

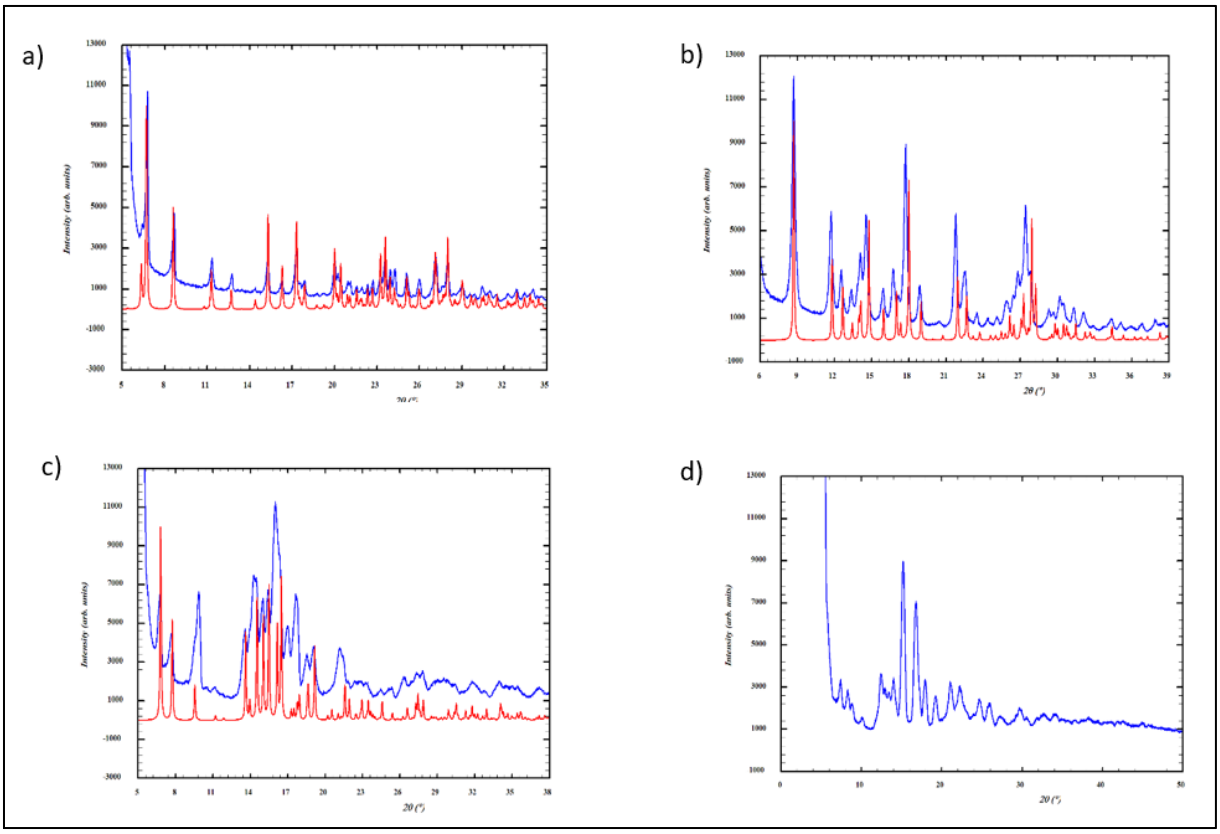

Figure S12: (a) Overlay of the experimental XRPD pattern of the commercial diclofenac sodium (blue) and the calculated pattern of anhydrous crystalline form of diclofenac sodium (code CSD AVASEY); (b) Overlay of the experimental XRPD pattern of the commercial piroxicam (blue) and the calculated pattern of polymorph I (code CSD BIYSEH14); (c) Overlay of the experimental XRPD pattern of the commercial dexamethasone 21-acetate (blue) and the calculated pattern of dexamethasone acetate monohydrate form I (code CSD UZAJUC); (d) Experimental XRPD pattern of the commercial bethamethasone 21-acetate.
